# Investigating language lateralization during phonological and semantic fluency tasks using functional transcranial Doppler sonography

**DOI:** 10.1080/1357650X.2014.914950

**Published:** 2014-05-29

**Authors:** Eva Gutierrez-Sigut, Heather Payne, Mairéad MacSweeney

**Affiliations:** ^a^ ESRC Deafness, Cognition and Language Research Centre, University College London, London, UK; ^b^ Institute of Cognitive Neuroscience, University College London, London, UK

**Keywords:** fTCD, Language lateralization, Phonological fluency, Semantic fluency, Word generation

## Abstract

Although there is consensus that the left hemisphere plays a critical role in language processing, some questions remain. Here we examine the influence of overt versus covert speech production on lateralization, the relationship between lateralization and behavioural measures of language performance and the strength of lateralization across the subcomponents of language. The present study used functional transcranial Doppler sonography (fTCD) to investigate lateralization of phonological and semantic fluency during both overt and covert word generation in right-handed adults. The laterality index (LI) was left lateralized in all conditions, and there was no difference in the strength of LI between overt and covert speech. This supports the validity of using overt speech in fTCD studies, another benefit of which is a reliable measure of speech production.

It has been well established that the left hemisphere plays a critical role in language processing in the majority of the population (Hellige, 1993). What is less well established is the extent to which individuals are consistent in their hemispheric lateralization across different language domains and across different task demands. The current study uses functional transcranial Doppler sonography (fTCD) to assess hemispheric lateralization during phonological and semantic fluency tasks requiring both overt and covert word generation.

fTCD was originally used as a clinical technique for determining blood flow velocity in the main cerebral arteries supplying the brain (Aaslid, 1987). Recently, this has become an effective way of investigating lateralization in healthy participants by measuring blood flow velocity simultaneously from bilateral probes while participants perform a cognitive task. fTCD is non-invasive and fast and shows high test–retest reliability (Knecht, Deppe, Ebner, et al., 1998). It shows high concordance with the Wada technique (Knake et al., 2003; Knecht, Deppe, Ringelstein, et al., 1998; Rihs et al., 1995) and functional magnetic resonance imaging (fMRI; Deppe et al., 2000; Somers et al., 2011), both in terms of proportions of participants measured as left lateralized and in terms of correlations with laterality indices (LIs).

To date, numerous studies have reported robust left hemisphere lateralization in right-handers using a phonological fluency task (e.g., Knecht et al., 1996, 2000, 2001; Lust, Geuze, Groothuis, & Bouma, 2011; Rosch, Bishop, & Badcock, 2012). This task is also referred to in the fTCD literature generically as a “word generation” task. Due to the characteristics of our experimental design, here we use the terms *phonological fluency* and *semantic fluency* for clarity.

The consistency of findings using a phonological fluency task has led to it becoming the gold standard task for assessing language lateralization with fTCD. In the phonological fluency task, the participant is presented with a series of letters one at a time and asked to generate as many words beginning with the letter as possible within a given time. Using this task, between 82% and 92.5% of right-handed participants are reported as showing left hemisphere dominance, while only 7.5–9.5% show right hemisphere dominance (Knecht et al., 2000, 2001). The phonological fluency task is usually performed covertly, that is, participants think of the words beginning with the target letter. Task adherence is usually assessed in one of two ways: participants either tap on the space bar each time they think of a new word (Krach & Hartje, 2006; Stroobant, Buijs, & Vingerhoets, 2009) or are required to verbally report some of the generated words in a later “report” period (see Badcock, Nye, & Bishop, 2012; Whitehouse & Bishop, 2009).

Although the covert phonological fluency task has proved to be remarkably effective in the assessment of language lateralization, dependence upon this task alone is unlikely to provide a complete pattern of language lateralization. Here we examine the potential benefits of using an *overt* word generation task and examine the use of a fluency task in a domain other than phonology: semantics.

## COVERT VERSUS OVERT TASKS

The extensive use of *covert* word generation tasks in fTCD studies examining language lateralization has been driven by the wish to minimize movement and potential auditory feedback artefacts in the TCD signal (e.g., Bishop, Watt, & Papadatou-Pastou, 2009; Knecht et al., 1996; Stroobant et al., 2009). However, covert tasks can be problematic for young children or special populations, where there may be a concern as to whether participants are accurately following the instructions. One possibility is to ask participants to whisper their response (Lust et al., 2011; Vingerhoets & Stroobant, 1999); however, even this may be difficult for young children. The desire to use fTCD with children has led to the development of appropriate *overt* production tasks (e.g., Bishop et al., 2009; Lohmann et al., 2005). For example, Bishop et al. (2009) required right-handed children and adults to overtly describe pictures and video animations. They demonstrated left hemisphere lateralization during these tasks. This suggests that hemispheric dominance for language can indeed be evaluated with fTCD using tasks that require overt speech. However, the overt speech tasks used in the fTCD literature to date do not allow for strict control over the output produced by the participant. That is, unlike the phonological fluency task used in the majority of previous *covert* fTCD studies, picture and animation description require extensive additional linguistic and cognitive processes, including semantic and syntactic processing (Bishop et al., 2009). No previous study, to our knowledge, has directly compared strength of lateralization when the same task is performed covertly versus overtly. In the current study we directly compare covert and overt word generation during word fluency tasks (phonological and semantic). This allows us to directly evaluate the impact of overt versus covert speech production on hemispheric lateralization. Bilateral motor cortices are necessarily involved in overt, more than covert, speech production (Price, 2010). One possibility therefore is that covert speech is more strongly left lateralized than overt speech.

Overt speech production tasks are not only easier than covert tasks for children and special population but also permit a more reliable assessment of the relationship between task performance and strength of lateralization. Studies to date that have used covert production have either not reported correlations between lateralization indices and number of words generated (e.g., Deppe et al., 2000) or have reported non-significant correlations (e.g., Knecht et al., 2000; Krach & Hartje, 2006; Stroobant et al., 2009). One possibility is that the lack of such a correlation is, at least in part, due to the indirect measure of the number of words produced during the covert period. This is either assessed at the same time as covert generation, by requesting a button press to represent the generation of a new word (Stroobant et al., 2009) or by the number of words reported *at the end* of the silent generation period (Badcock et al., 2012; Deppe et al., 2000; Knecht et al., 1996). Lust et al. (2011) used an *overt* phonological fluency task while recording fTCD data but, again, reported no relationship between the number of words produced and the strength of lateralization. However, the participants in that study were given fluency instructions from the Controlled Oral Word Association Test (Ruff, Light, Parker, & Levin, 1996), which do not permit repetitions, proper nouns or numbers as responses. Perhaps not surprisingly, under these conditions participants only produced a mean of five words in a response window of 20 s. We adopt a more lenient approach to scoring output in the current study, which we argue more accurately reflects the natural fluency of participants.

By measuring lateralization as well as the behavioural responses during overt word generation, we will test the hypothesis that there is a positive correlation between the number of items produced and the strength of hemispheric lateralization during a word generation task.

## PHONOLOGICAL VERSUS SEMANTIC TASKS

Another factor likely to influence the degree of hemispheric lateralization is the language domain tested. The literature suggests that some tasks are more likely to lead to robust left hemisphere lateralization than others. Tasks that tax phonological skills, such as rhyme generation (Krach & Hartje, 2006) and the gold standard phonological fluency task (Deppe et al., 2000; Knecht, Deppe, Ebner, et al. 1998; Knecht et al., 2000), appear to drive left hemisphere lateralization to a greater extent than less phonological language tasks.

In order to provide a more comprehensive view of lateralization of language processing Stroobant et al. (2009) explored a range of language tasks within the same participants. The authors measured fTCD signal during: (1) covert phonological fluency; (2) sentence construction (from a series of words presented in a mixed order); (3) reading aloud fragments of natural text and (4) semantic decision (deciding which of three words was not synonymous with the others). All tasks were left lateralized at a group level; however, the percentage of left-lateralized participants differed depending on the task: sentence construction (90%), phonological fluency (80%), reading (73%) and semantic decision (67%).

Critically, the semantic task used by Stroobant et al. (2009) was a comprehension task. Other studies in the literature that have assessed semantic processing have also used more receptive tasks as opposed to the production tasks typically used to assess phonological processing (Rihs et al., 1995; Vingerhoets & Stroobant, 1999). Tasks used to assess semantic processing include listening to a short passage and answering multiple choice questions and listening to a word definition and then generating the target word (Badcock et al., 2012). Production tasks tend to produce stronger left hemisphere lateralization than more receptive tasks as measured with fTCD (Badcock et al., 2012; Buchinger et al., 2000; Stroobant, Van Boxstael, & Vingerhoets, 2011) and fMRI (Gaillard et al., 2004). Therefore, the extent to which the strong left hemispheric lateralization observed for phonological tasks is due to the use of *phonological skills* versus *speech production* (whether this is overtly or covertly produced) is unclear. Here we avoid this confound between language domain and language task, by using a word fluency task to assess *both* phonological and semantic processing.

In summary, the current study compares hemispheric lateralization during phonological and semantic fluency tasks during both covert and overt speech production. We predict a greater number of trials to be excluded from the “overt” condition than the “covert” condition because of artefacts during measurement. Nevertheless, we include a sufficient number of trials to allow us to examine our questions of interest. First, we test the hypothesis that the *strength* of lateralization index, as measured by fTCD, is modulated by whether covert or overt production is required. If overt speech production is largely driven by activation in bilateral motor cortices, then we would observe a lower LI during overt than covert speech. Second, we test the hypothesis that there is a positive relationship between the strength of LI and the number of words produced. The inclusion of an overt speech condition allows a more accurate assessment of this potential relationship, since both are direct measures, which are taken concurrently. Finally, contrasting phonological and semantic fluency tasks allow us to examine the strength of hemispheric dominance across different language domains. On the basis of previous studies, we predict a stronger lateralization index for phonological than semantic fluency.

## METHODS

### Design

We used a 2 (*production type*: covert vs. overt) ×2 (*language task*: phonological vs. semantic) design. The resulting four conditions were presented in separate blocks, the order of which was counterbalanced across participants: phonological-covert, phonological-overt, semantic-covert and semantic-overt.

### Participants

A total of 29 participants (16 females) were recruited from the Institute of Cognitive Neuroscience volunteer database. Twenty one were students at University College London (UCL), three were recent graduates from UCL and the remaining five participants were from the local community. The mean age of the participants was 27.2 years (range 19–46 years), and all had English as their first language. No participants reported a history of neurological disorders or language-related problems. Participants were all right handed as assessed by an abridged version of the Edinburgh Handedness Inventory (Oldfield, 1971). The questionnaire comprised 10 questions about handedness and 4 questions related to footedness for regular activities such as writing or kicking a ball. The mean number of activities performed with the right hand was 9.46 out of 10. The mean number of activities performed exclusively with the right foot was 2.7 out of 4. None of the participants reported any activity done exclusively with the left hand or foot. Due to insonation difficulties, it was not possible to find a good signal in three participants in two of the experimental conditions. These participants were therefore excluded from the analyses. The mean age of the remaining 26 participants was 27.2 years (range 19–46 years), with 14 females.

### Stimuli

#### Phonological fluency

Ten letters that have been reliably used in a number of previous phonological fluency studies were chosen (A, B, C, F, H, M, O, S, T and W). Each letter was presented twice within each condition: covert phonological fluency/overt phonological fluency. Thus, each condition consisted of 20 trials, which were presented in a pseudo-randomized order to ensure that all 10 letters had been presented once before it was repeated.

#### Semantic fluency

The following categories were chosen: Farm Animals, Zoo Animals, Vegetables, Fruits, Drinks, Colours, Sports, Pets, Tools and Transport. These categories were repeated twice within each of the semantic fluency task blocks, resulting in 20 trials per block, which were presented in a pseudo-randomized order (as above).

### Procedure

Ethical approval for the study was obtained from the UCL Research Ethics Committee. All participants gave written informed consent before the study. The whole session, including set up time, lasted approximately 90 min. Each block was preceded by two practice trials showing categories or letters that were not used in the experimental blocks.

#### Covert blocks

**Figure 1.**
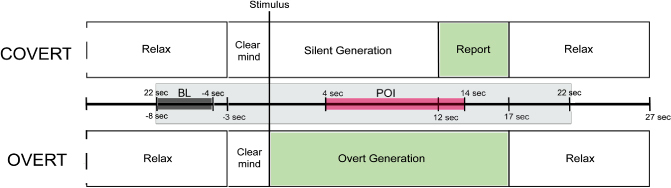
Schematic diagram of timing of events within covert and overt trials.

Each trial began with a 3-s preparation period during which “clear your mind” was displayed on the screen and participants were instructed to focus on the screen (see Figure 1). The cue, either a single letter or a semantic category, was then displayed for 12 s. Participants were asked to silently generate as many words as possible beginning with the letter/belonging to the category displayed on the screen. To ensure compliance with the task, at the end of the covert phase, participants were asked to overtly report as many of the words they had generated as possible. This “report” period lasted for 5 s. A short report period was used to replicate the timing for the report period used in the previous fTCD studies of covert word generation. In addition, this report period ensured the same duration of overall length of trial between overt and covert conditions. The report phase was followed by a “relax” prompt, which appeared for 10 s. Participants were instructed to use the “relax” period to imagine a peaceful scene. The overall trial duration was 30 s, which is shorter than many previous studies of word generation (see e.g., Knecht, Deppe, Ringelstein, et al., 1998). Nonetheless, satisfactory baseline measures were established for all conditions. In addition, the shorter trial duration is more enjoyable for participants than longer trials.

Stimuli were presented using Cogent toolbox (http://www.vislab.ucl.ac.uk/cogent) for MATLAB (Mathworks Inc., Sherborn, MA). Triggers time locked to the onset of the stimulus were sent from the presentation PC to the Doppler Box set-up.

#### Overt blocks

The overt blocks proceeded in exactly the same way as the covert ones, except that the participants reported the words aloud as soon as the stimulus had been presented (see Figure 1). In this case, the stimulus was displayed for 17 s.

### fTCD recording and processing

Blood flow velocity through the left and right MCAs was examined using a Doppler ultrasonography device (DWL DopplerBox; manufactured by DWL Elektronische Systeme, Singen, Germany). Two 2-MHz transducer probes were mounted on a flexible headset and placed at each temporal skull window.

Data analysis was carried out with dopOSCCI, a custom MATLAB programme written for analysing fTCD group data (Badcock, Holt, Holden, & Bishop, 2012). Analysis involved down-sampling of the data from 100 Hz to 25 Hz, normalization of left and right channel values, heart cycle integration and artefact rejection. Epochs with values less than 70% or greater than 120% of the average blood flow velocity were excluded from the analyses. Epochs were segmented from −8 to 22 s relative to stimulus presentation. All data points were baseline corrected by subtracting the blood flow velocity during a period of inactivity −8 to −4 s before the onset of stimulus. The period of interest (POI) was set from 4 to 14 s after the onset of stimulus. To ensure that blood flow for the baseline period was always calculated from resting level, the first trial of the block was not included in the analyses. This resulted in 19 analysed trials per block. LIs were calculated for each participant separately, for each of the four conditions. For each participant, the maximum peak left–right difference within the POI was identified. The 2-s measurement window was centred on this maximum. The LI was defined as the average of the left minus right differences within this 2-s window.

### Behavioural responses

Participants' behavioural responses were recorded for scoring offline. In the phonological fluency conditions, items were considered correct if they began with the target letter. Words that started with the target letter “sound” were also classed as correct (e.g., phone for /f/ was allowed). In the semantic conditions, items semantically linked to the category were allowed. However, describing phrases, for example, “good for you” in response to the target “vegetables”, were counted as errors.

## RESULTS

### Artefact rejection of fTCD epochs

In order to investigate whether overt speech led to more artefacts during recording, we first analysed the number of epochs remaining for each participant after the artefact rejection procedure (see Methods). A repeated-measures analysis of variance (ANOVA) revealed a significant effect of production type on the number of epochs accepted, *F*(1, 25) = 6.8, *MSE* = 6.67, *p* > .015, 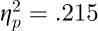, with fewer epochs accepted in the overt than covert conditions (mean across fluency tasks: 14.4 [overt; min = 5, max = 19] vs. 15.8 [covert; min = 4, max = 19]). There was no main effect of *language task* [*F <* 1, 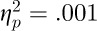] and no significant interaction [*F* < 1, 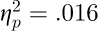].

Participants with fewer than eight usable epochs in any condition (based on artefact rejection parameters in the Methods) were excluded from further analyses. Four participants were excluded on this basis. Half of these exclusions were due to artefacts in overt conditions and half due to artefacts in covert conditions. Therefore, 22 participants were included in the rest of the analyses.

### Behavioural data for participants included in fTCD analyses

Table 1 shows the average number of words reported for each trial in each of the four conditions for the 22 participants with the required number of acceptable epochs per block. A repeated-measures ANOVA on the number of correctly produced words revealed a main effect of production type, *F*(1, 21) = 217.6, *MSE* = 1.60, *p* < .0001, 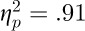, with more words produced during the overt than covert task (mean 8.92 vs. 4.92 words per trial). This is as expected, given the difference in response time windows between conditions.

**TABLE 1:** Mean number of words generated in each condition

Production type	Language task	Mean number of words per trial	SD	Minimum	Maximum
Covert	Phonological	4.6	1.01	3	8
	Semantic	5.3	1.08	4	8
Overt	Phonological	8.5	1.72	5	12
	Semantic	9.3	2.40	3	13

Of greater interest, the main effect of *language task* was also significant, *F*(1, 21) = 12.3, *MSE* = .98, *p* < .001, 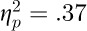, with more words produced during the semantic than phonological task (mean 7.4 vs. 6.7 words per trial). There was no significant interaction (*F* < 1, 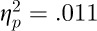).

### Reliability of fTCD data

In order to assess the reliability of the fTCD data, we conducted split half reliability analyses on each condition separately. Odd and even epochs were correlated within the semantic overt (*r* = .61, *p* = .003) and semantic covert (*r* = .42, *p* = .05) conditions. However, although the trend was in the same direction, the relationship did not reach significance for the phonological covert (*r* = .38, *p* = .08) or phonological overt (*r* = .32, *p* = .14) conditions.

### Mean LI and percentage of subjects left lateralized

A positive LI is indicative of left lateralization and a negative LI of right lateralization. One-sample *t*-tests were used to assess whether the LI value was significantly left or right lateralized for each participant and condition. When the one-sample *t*-test did not reach significance, participants were considered as “low lateralized” for that condition. This situation has also been referred to by others in the field as “bilateral lateralization” (e.g., Badcock et al., 2012; Bishop et al., 2009).

**Figure 2.**
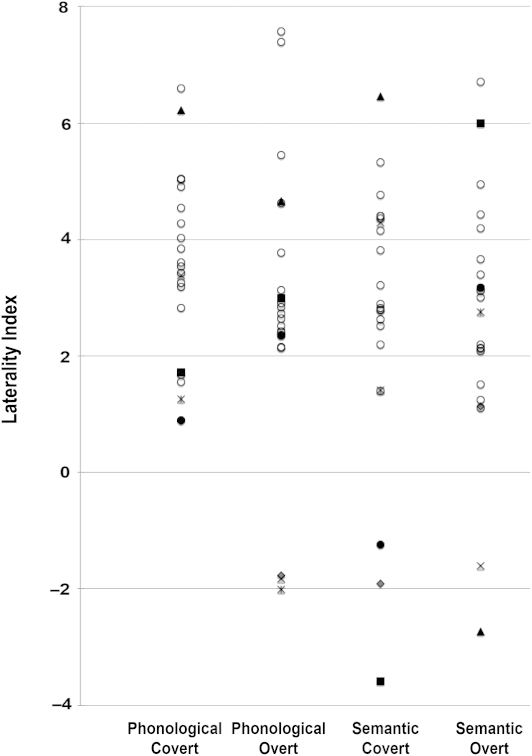
Individual LI scatterplots for each condition. The LIs for atypical individuals in any of the four conditions are shaped-coded. Each shape consistently represents each of these six participants across conditions.

In all conditions, group-averaged LIs were positive. In addition, one-sample *t*-tests showed that each of the four conditions were significantly different to zero and can be considered left lateralized at a group level (see Table 2 and Figure 2). Although at the group level each condition was clearly left lateralized, not all participants in the group showed this pattern. Table 2 shows the number of participants who showed low laterality (not significantly different to zero) or were right lateralized (negative LI, significantly different to zero) in each condition. This variability is also displayed in Figure 2. This illustrates the LIs for each participant in each condition. The six participants who had a negative LI in any of the four conditions are shape coded. One participant (black triangle) was right lateralized in the semantic overt task while strongly left lateralized in the other three conditions. A second participant (asterisk) was right lateralized in the phonological overt and low lateralized in phonological and semantic covert but left lateralized in the semantic overt task. A third participant (black circle) was considered low in both covert tasks, although with negative LI in semantic covert and left lateralized in both overt tasks. A fourth participant (grey diamond) was considered low lateralized in all conditions, showing negative LIs in phonological overt and semantic covert tasks. A fifth participant (cross) was left lateralized for both covert tasks but right lateralized for both overt ones. Finally, a sixth participant (black square) was right lateralized in the semantic covert, low lateralized in the phonological covert and strongly left lateralized in both overt conditions. Detailed visual inspection of individual trials from these participants did not show more artefacts or signal noise for them than for the rest of the participants.

**TABLE 2:** The left side of the table shows the mean LI values and group one-sample *t*-tests for each condition and the right side of the table shows the number (percentage between brackets) of participants left, right and “low” lateralized (fTCD recording and processing) in each condition

		LI						
Production type	Language task	Mean	SD	t	p	Left lateralized, n (%)	Right lateralized, n (%)	Low laterality, n (%)
Covert	Phonological	3.4	1.6	10.0	<.001	18 (82%)	0	4 (18%)
	Semantic	2.7	2.4	5.3	<.001	17 (77%)	1 (5%)	4 (18%)
Overt	Phonological	2.8	2.4	5.3	<.001	17 (77%)	2 (10%)	3 (13%)
	Semantic	2.6	2.2	5.7	<.001	18 (82%)	1 (5%)	3 (13%)

### LI differences between conditions

**Figure 3.**
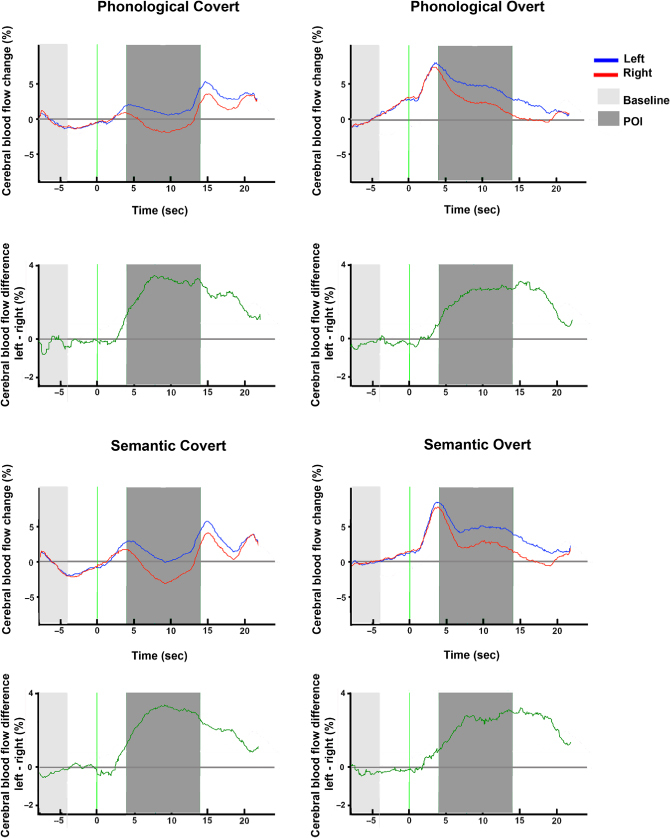
Average of participants' baseline-corrected cerebral blood flow velocity for the left (blue line) and right (red line) channels for each of the four conditions. The difference between left and right channels is shown below each condition. The baseline and periods of interest for the computation of LIs are shown in light and dark grey, respectively. [To view this figure in colour, please see the online version of this journal.]

A repeated-measures ANOVA showed no differences in LI strength between conditions. The main effects of *production type*, *F*(1, 21) = .48, *MSE* = 6.37, *p* > .1, 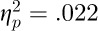, and *language task*, *F*(1, 21) = 2.85, *MSE* = 1.52, *p* > .1, 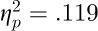, as well as the interaction (*F* < 1, 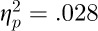) were not significant. Figure 3 shows the average of participants' cerebral blood flow velocity for the left and right channels for each condition.

### Relationship between LI and number of words generated

**Figure 4.**
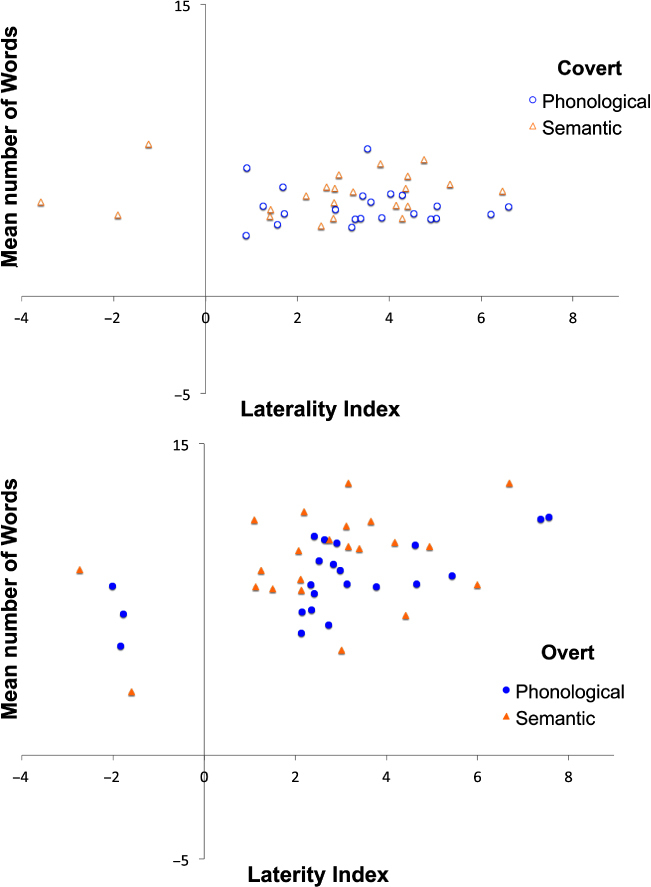
Scatterplots showing relationships between the LIs and the number of words produced. This relationship was not significant during covert generation (top panel) but was significant during phonological overt generation (bottom panel).

Given the difference in the response time window between overt and covert trials, correlations between the number of words generated and LI during each condition were examined separately (see Figure 4). For covert generation there was no significant correlation between LI and the number of words produced in the phonological (*r* = −.08, *p* > .1) or in the semantic condition (*r* = −.11, *p* > .1). However, there was a significant correlation between strength of LI and the number of words produced in the overt phonological condition (*r* = .64, *p* = .001), yet the correlation between words produced and LI in the overt semantic condition just failed to reach significance (*r* = .4, *p* = .063).

In order to avoid distortion of the correlations from participants who demonstrated “atypical” language lateralization (Cai, Van der Haegen, & Brysbaert, 2013; Illingworth & Bishop, 2009; Whitehouse & Bishop, 2008), participants who had LI values lower than 0, and therefore a right hemisphere bias, in any of the conditions were excluded from the analyses. After excluding these six participants (see Figure 2 for details), a similar pattern of relationship was observed. The correlation between number of words produced and strength of LI was not significant during the covert phonological task (*r* = −.08, *p* > .1), while this relationship did just reach significance in the semantic covert task (*r* = .51, *p* = .05). For the overt speech conditions, there was again a positive correlation between the strength of LI and the number of words produced in the phonological condition (*r* = .61, *p* = .01), but not in the semantic condition (*r* = .23, *p* > .1).

### Relationship between strength of LI across conditions

**Figure 5.**
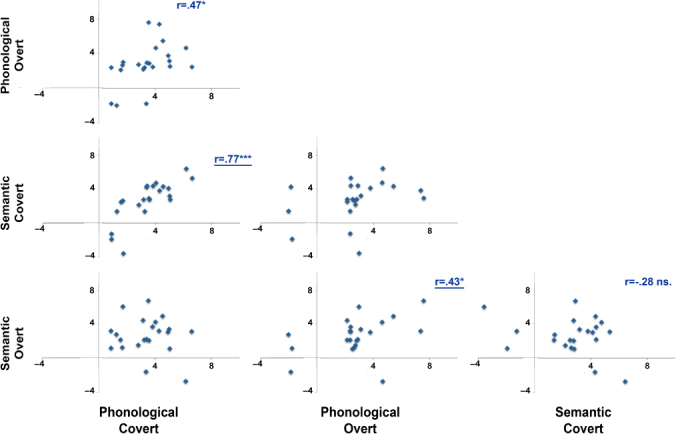
Individual LI scatterplots between conditions. Pearson correlations are displayed for relevant relationships (*p < .05, ***p < .0001, *ns* = non-significant). Pearson's *r* is underlined for those correlations that are significant after exclusion of “low” or right-lateralized participants.

Correlations between the mean LI for each of the four conditions (see Figure 5) showed that LIs for both language tasks (phonological and semantic) were correlated for the covert (*r* = .77, *p* < .0001) and the overt (*r* = .43, *p* = .047) tasks. In addition, both phonological production types (covert and overt) were correlated (*r* = .47, *p* = .03). Semantic production types, however, were not significantly correlated (*r* = −.28, *p* > .1).

Again, we ran the same analyses after excluding those participants with an LI lower than zero in any of the conditions. Again, the correlations between LIs for both language tasks (phonological and semantic) were significant for the covert (*r* = .61, *p* = .011) and the overt (*r* = .67, *p* = .0075) tasks. However, correlations across production type were not significant: phonological covert and overt (*r* = .16, *p* > .1); semantic covert and overt (*r* = .34, *p* > .1).

## DISCUSSION

We examined hemispheric lateralization of processing across different language tasks, using fTCD. Our design allowed us to examine the influence of overt versus covert speech production and phonological versus semantic processing on the strength of the TCD signal. These two factors have been confounded in many previous studies, which have often used production tasks to examine phonological processing and comprehension tasks to examine semantic processing.

The mean LI for all four conditions was positive and can therefore be considered left lateralized at a group level. In addition, at the individual level the majority of participants were categorized as left lateralized in each condition. However, the strength of the LIs was not influenced by production type or language task. Nor was there an interaction between the two factors. Nevertheless, these null findings, in combination with additional correlational analyses, lead us to some important conclusions regarding the usefulness and sensitivity of TCD as a research tool.

### Covert versus overt generation

As we predicted, overall a greater number of epochs were rejected from the overt than the covert speech conditions. However, importantly when applying inclusion criteria of eight or more usable epochs in each condition, the same number of participants were rejected from overt and covert conditions. This suggests, at least with the POI that we selected in these analyses, that sufficient trials of good quality data can be collected when overt speech is required. In addition, we found that covert and overt word generation did not differ in terms of strength of LI, and the same number of participants were categorized as left lateralized during each mode of production (80% in both covert and overt conditions). Thus, our findings contribute to the increasing body of studies that have validated the use of overt speech production tasks to measure language lateralization using fTCD (e.g., Bishop et al., 2009; Lohmann et al., 2005). Our findings further previous studies by directly contrasting overt and covert speech production during the performance of the same language task (word generation during a fluency task).

fMRI studies have demonstrated that although there is extensive activation of bilateral motor regions during overt speech production (e.g., Murphy et al., 1997; Price, 2012; Riecker et al., 2005; Stewart, Walsh, Frith, & Rothwell, 2001), prefrontal cortex activation is typically left lateralized (Riecker et al., 2005; Terumitsu, Fujii, Suzuki, Kwee, & Nakada, 2006). Our finding of no difference in strength of LI between overt and covert conditions supports the suggestion that the blood velocity changes, as measured by fTCD, are driven predominantly by pre-motor processes. Although the LIs did not differ between overt and covert conditions, the pattern of correlations between LIs in the different conditions suggests subtle differences between the two production types. There were significant positive correlations between both overt conditions and covert conditions, but not *across* production types. Also in line with previous studies, we found no correlation between the number of words generated in the report phase of the covert trials and the strength of LI, measured during the covert generation period (Badcock et al., 2012; Knecht et al., 2001; Stroobant et al., 2009). However, the lack of correlation between these non-contemporaneous measures is most likely due to a ceiling effect on the number of items that can be reported within the short time window. We argue this on the basis of one of our novel findings from the current study: that of a positive correlation between the number of words generated in the *phonological overt* trials and the strength of LI. This relationship was significant when we considered the group as a whole and also when we excluded those considered to have “low” or right hemisphere lateralization. Accounting for this relationship in terms of greater primary motor demands alone seems unlikely, given that there are no overall differences in LI between overt and covert trials. Rather it is more likely that this relationship reflects the increasing demands on pre-motor cortex as more words are generated. The role of language domain in this pattern will now be considered.

### Phonological versus semantic processing

The overall strength of LI did not differ between phonological and semantic word generation. This lack of difference in LI could be related to the fact that here phonological and semantic processing were tested using production tasks. Previous fTCD studies have typically confounded the use of receptive versus production tasks with linguistic domain. This null finding emphasizes the fact that task requirements should be taken into account when contrasting tasks across language domains. Even though there was no significant difference in LIs between phonological and semantic tasks, as with the overt/covert speech contrast, correlations suggested subtle differences between the two language domains, despite very similar task requirements.

In accordance with previous behavioural studies, participants produced more words during the semantic than phonological fluency task (Crowe, 1998; Hurks et al., 2006; Monsch et al., 1994). However, the correlation between number of words produced and strength of LI was significant only in the phonological condition. One possible interpretation of this is that phonological search is more dependent on pre-motor processes, measured by fTCD, than semantic search.

In summary, we found no difference in strength of LI between overt and covert word generation measured using fTCD. This suggests that during the current test conditions, the fTCD signal was not greatly influenced by motor processes, but was most likely driven by pre-motor activity as well as linguistic and cognitive processes. Our data demonstrate that *overt* word generation can be successfully used to assess language lateralization using fTCD. There are a number of reasons in favour of using overt word generation tasks in fTCD studies. First, overt word generation does not require many of the additional cognitive processes that are involved in a covert generation task with a later response period, such as response selection, short-term memory and evaluation of acceptable responses for reporting (see, e.g., Badcock et al., 2012). Second, an accurate measure of the behavioural response can be established. Third, the behavioural response is measured at the *same time* as the physiological response used to calculate the LIs. We have demonstrated that accurate measurement of the behavioural response and simultaneous recording of the LI allows correlational analyses that may provide a more complete picture of fTCD signal change, than simply considering effects of task manipulations in the absence of individual differences in behaviour.

Although the fTCD methodology is extremely basic in contrast to fMRI or other neuroimaging approaches, when used appropriately it can be used to provide more than a very broad description of “language lateralization”. Our results show that while language subdomains do not give rise to significantly different LIs, patterns in the data can be distinguished using this technique, especially when accurate measures of behaviour are collected concurrently. As such this simple, non-invasive tool lends itself ideally to further explore different aspects of language processing in children and special populations.
